# voom: precision weights unlock linear model analysis tools for RNA-seq read counts

**DOI:** 10.1186/gb-2014-15-2-r29

**Published:** 2014-02-03

**Authors:** Charity W Law, Yunshun Chen, Wei Shi, Gordon K Smyth

**Affiliations:** 1 Bioinformatics Division, The Walter and Eliza Hall Institute of Medical Research, 1G Royal Parade, Parkville, Victoria 3052, Australia; 2 Department of Medical Biology, The University of Melbourne, Parkville, Victoria 3010, Australia; 3 Department of Computing and Information Systems, The University of Melbourne, Parkville, Victoria 3010, Australia; 4 Department of Mathematics and Statistics, The University of Melbourne, Parkville, Victoria 3010, Australia

## Abstract

New normal linear modeling strategies are presented for analyzing read counts from RNA-seq experiments. The voom method estimates the mean-variance relationship of the log-counts, generates a precision weight for each observation and enters these into the limma empirical Bayes analysis pipeline. This opens access for RNA-seq analysts to a large body of methodology developed for microarrays. Simulation studies show that voom performs as well or better than count-based RNA-seq methods even when the data are generated according to the assumptions of the earlier methods. Two case studies illustrate the use of linear modeling and gene set testing methods.

## Background

Gene expression profiling is one of the most commonly used genomic techniques in biological research. For most of the past 16 years or more, DNA microarrays have been the premier technology for genome-wide gene expression experiments, and a large body of mature statistical methods and tools has been developed to analyze intensity data from microarrays. This includes methods for differential expression analysis [1-3], random effects [4,5], gene set enrichment [6], gene set testing [7,8] and so on. One popular differential expression pipeline is that provided by the limma software package [9]. The limma pipeline includes linear modeling to analyze complex experiments with multiple treatment factors, quantitative weights to account for variations in precision between different observations, and empirical Bayes statistical methods to borrow strength between genes.

Borrowing information between genes is a crucial feature of the genome-wide statistical methods, as it allows for gene-specific variation while still providing reliable inference with small sample sizes. The normal-based empirical Bayes statistical procedures can adapt to different types of datasets and can provide exact type I error rate control even for experiments with a small number of replicate samples [3].

In the past few years, RNA-seq has emerged as a revolutionary new technology for expression profiling [10]. One common approach to summarize RNA-seq data is to count the number of sequence reads mapping to each gene or genomic feature of interest [11-14]. RNA-seq profiles consist therefore of integer counts, unlike microarrays, which yield intensities that are essentially continuous numerical measurements. A number of early RNA-seq publications applied statistical methods developed for microarrays to analyze RNA-seq read counts. For example, the limma package has been used to analyze log-counts after normalization by sequencing depth [11,15-17].

Later statistical publications argued that RNA-seq data should be analyzed by statistical methods designed specifically for counts. Much interest has focused on the negative binomial (NB) distribution as a model for read counts, and especially on the problem of estimating biological variability for experiments with small numbers of replicates. One approach is to fit a global value or global trend to the NB dispersions [13,18,19], although this has the limitation of not allowing for gene-specific variation. A number of empirical Bayes procedures have been proposed to estimate the gene-wise dispersions [20-22]. Alternatively, Lund *et al*. [23] proposed that the residual deviances from NB generalized linear models be entered into limma’s empirical Bayes procedure to enable quasi-likelihood testing. Other methods based on over-dispersed Poisson models have also been proposed [24-26].

Unfortunately, the mathematical theory of count distributions is less tractable than that of the normal distribution, and this tends to limit both the performance and the usefulness of the RNA-seq analysis methods. One problem relates to error rate control with small sample sizes. Despite the use of probabilistic distributions, all the statistical methods developed for RNA-seq counts rely on approximations of various kinds. Many rely on the statistical tests that are only asymptotically valid or are theoretically accurate only when the dispersion is small. All the differential expression methods currently available based on the NB distribution treat the estimated dispersions as if they were known parameters, without allowing for the uncertainty of estimation, and this leads to statistical tests that are overly liberal in some situations [27,28]. This is true even of the NB exact test [18], which gives exact type I error rate control when the dispersion is known but which becomes liberal when an imprecise dispersion estimator is inserted for the known value. Quasi-likelihood methods [23] account for uncertainty in the dispersion by using an *F*-test in place of the usual likelihood ratio test, but this relies on other approximations, in particular that the residual deviances are analogous to residual sums of squares from a normal analysis of variance.

A related issue is the ability to adapt to different types of data with high or low dispersion heterogeneity. None of the empirical Bayes methods based on the NB distribution achieve the same adaptability, robustness or small sample properties as the corresponding methods for microarrays, due to the mathematical intractability of count distributions compared to the normal distribution.

The most serious limitation though is the reduced range of statistical tools associated with count distributions compared to the normal distribution. This is more fundamental than the other problems because it limits the types of analyses that can be done. Much of the statistical methodology that has been developed for microarray data relies on use of the normal distribution. For example, we often find it useful in our own microarray gene expression studies to estimate empirical quality weights to downweight poor quality RNA samples [29], to use random effects to allow for repeated measures on the same experimental units [4,5] or to conduct gene set tests for expression signatures while allowing for inter-gene correlations [7,8]. These techniques broaden the range of experimental designs that can be analyzed or offer improved interpretation for differential expression results in terms of higher level molecular processes. None of these techniques are currently available for RNA-seq analysis using count distributions.

For these reasons, the purpose of this article is to revisit the idea of applying normal-based microarray-like statistical methods to RNA-seq read counts. An obstacle to applying normal-based statistical methods to read counts is that the counts have markedly unequal variabilities, even after log-transformation. Large counts have much larger standard deviations than small counts. While a logarithmic transformation counteracts this, it overdoes the adjustment somewhat so that large log-counts now have smaller standard deviations than small log-counts. We explore the idea that it is more important to model the mean-variance relationship correctly than it is to specify the exact probabilistic distribution of the counts. There is a body of theory in the statistical literature showing that correct modeling of the mean-variance relationship inherent in a data generating process is the key to designing statistically powerful methods of analysis [30]. Such variance modeling may in fact take precedence over identifying the exact probability law that the data values follow [31-33]. We therefore take the view that it is crucial to understand the way in which the variability of RNA-seq read counts depends on the size of the counts. Our work is in the spirit of pseudo-likelihoods [32] whereby statistical methods based on the normal distribution are applied after estimating a mean-variance function for the data at hand.

Our approach is to estimate the mean-variance relationship robustly and non-parametrically from the data. We work with log-counts normalized for sequence depth, specifically with log-counts per million (log-cpm). The mean-variance is fitted to the gene-wise standard deviations of the log-cpm as a function of average log-count. We explore two ways to incorporate the mean-variance relationship into the differential expression analysis. The first is to modify limma’s empirical Bayes procedure to incorporate a mean-variance trend. The second method incorporates the mean-variance trend into a precision weight for each individual normalized observation. The normalized log-counts and associated precision weights can then be entered into the limma analysis pipeline, or indeed into any statistical pipeline for microarray data that is precision weight aware. We call the first method limma-trend and the second method voom, an acronym for ‘variance modeling at the observational level’. limma-trend applies the mean-variance relationship at the gene level whereas voom applies it at the level of individual observations.

This article compares the performance of the limma-based pipelines to edgeR [20,34], DESeq [13], baySeq [21], TSPM [25], PoissonSeq [26] and DSS [22], all of which are based on NB or over-dispersed Poisson distributions. Simulation studies show that the limma pipelines perform at least as well in terms of power and error rate control as the NB or Poisson methods even when the data is generated according to the probabilistic assumptions of the earlier methods. A key advantage of the limma pipelines is that they provide accurate type I error rate control even when the number of RNA-seq samples is small. The NB and Poisson based methods either fail to control the error correctly or are excessively conservative. limma-trend and voom perform almost equally well when the sequencing depths are the same for each RNA sample. When the sequencing depths are different, voom is the clear best performer.

Either voom or limma-trend give RNA-seq analysts immediate access to many techniques developed for microarrays that are not otherwise available for RNA-seq, including all the quality weighting, random effects and gene set testing techniques mentioned above. This article presents two case studies that demonstrate how voom can handle heterogeneous data and complex experiments as well as facilitating pathway analysis and gene set testing.

## Results

### Counts per million: a simple interpretable scale for assessing differential expression

We suppose that RNA-seq profiles (or *libraries*) are available for a set of *n* RNA samples. Each profile records the number of sequence reads from that sample that have been mapped to each one of *G* genomic features. A genomic feature can be any predefined subset of the transcriptome, for example a transcript, an exon or a gene. For simplicity, we will assume throughout this article that reads have been summarized by gene, so that the RNA-seq profiles give the number of reads from each sample that have been mapped to each gene. Typically *G* is large, in the tens of thousands or more, whereas *n* can be as low as three. The total number of mapped reads (*library size*) for each sample might vary from a few hundred thousand to hundreds of millions. This is the same context as assumed by a number of previous articles [13,18,20,21,34].

The number of reads observed for a given gene is proportional not just to the expression level of the gene but also to its gene transcript length and to the sequencing depth of the library. Dividing each read count by the corresponding library size (in millions) yields counts per million (cpm), a simple measure of read abundance that can be compared across libraries of different sizes. Standardizing further by transcript length (in kilobases) gives rise to reads per kilobase per million (rpkm), a well-accepted measure of gene expression [35]. In this article we will work with the simpler cpm rather than rpkm, because we are interested in relative changes in expression between conditions rather than absolute expression.

This article treats log-cpm as analogous to log-intensity values from a microarray experiment, with the difference that log-cpm values cannot be treated as having constant variances. Differences in log-cpm between samples can be interpreted as log-fold-changes of expression. The counts are augmented by a small positive value (a half of one read) to avoid taking the logarithm of zero. This ensures no missing log-cpm values and reduces the variability at low count values.

### Log-cpms have stabilized variances at high counts

Probability distributions for counts are naturally heteroscedastic, with larger variances for larger counts. It has previously been argued that the mean-variance relationship for RNA-seq counts should be approximately quadratic [34]. This leads to the conclusion that the coefficient of variation (CV) of RNA-seq counts should be a decreasing function of count size for small to moderate counts but for larger counts should asymptote to a value that depends on biological variability. Specifically, the squared CV of the counts should be roughly

1/λ+ϕ

where *λ* is the expected size of the count and *ϕ* is a measure of biological variation [34]. The first term arises from the technical variability associated with sequencing, and gradually decreases with expected count size, while biological variation remains roughly constant. For large counts, the CV is determined mainly by biological variation.

**Figure 1:**
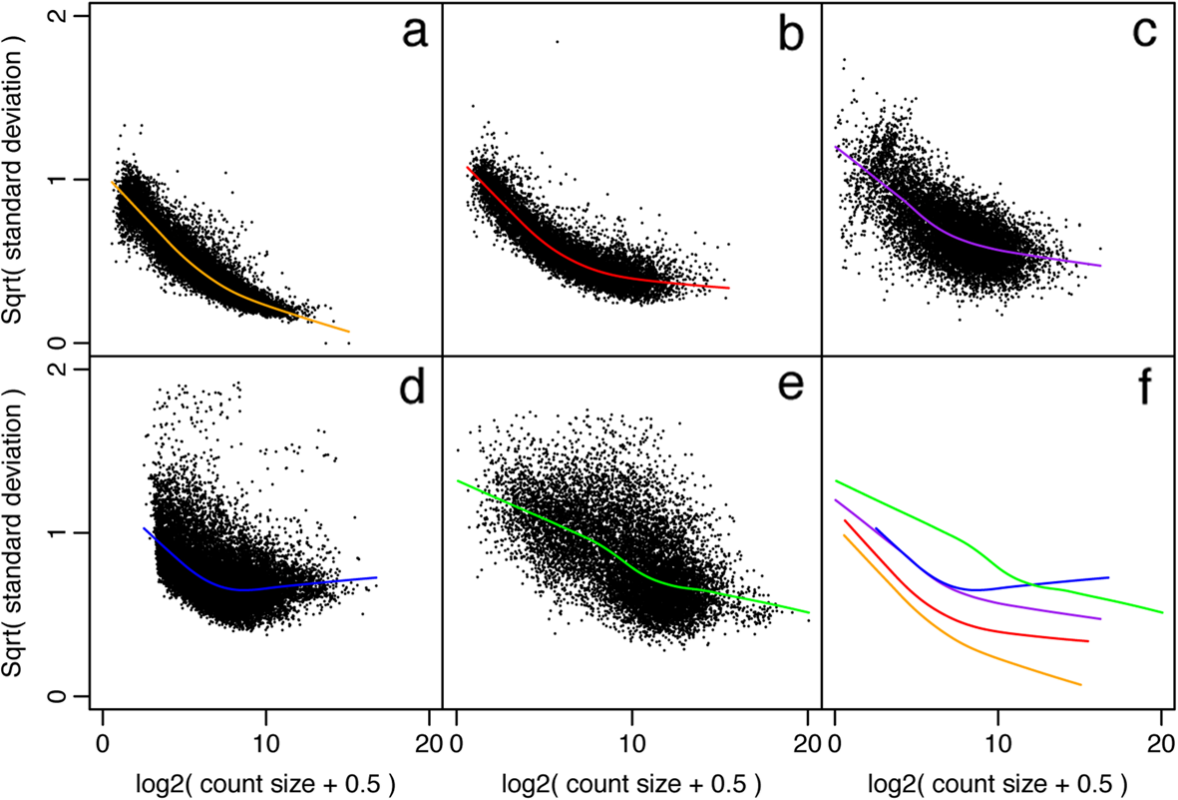
**Mean-variance relationships.** Gene-wise means and variances of RNA-seq data are represented by black points with a LOWESS trend. Plots are ordered by increasing levels of biological variation in datasets. **(a)** voom trend for HBRR and UHRR genes for Samples A, B, C and D of the SEQC project; technical variation only. **(b)** C57BL/6J and DBA mouse experiment; low-level biological variation. **(c)** Simulation study in the presence of 100 upregulating genes and 100 downregulating genes; moderate-level biological variation. **(d)** Nigerian lymphoblastoid cell lines; high-level biological variation. **(e)***Drosophila melanogaster* embryonic developmental stages; very high biological variation due to systematic differences between samples. **(f)** LOWESS voom trends for datasets (a)–(e). HBRR, Ambion’s Human Brain Reference RNA; LOWESS, locally weighted regression; UHRR, Stratagene’s Universal Human Reference RNA.

A simple linearization calculation suggests that the standard deviation of the log-cpm should be approximately equal to the CV of the counts (see Materials and methods). Examination of a wide range of real datasets confirms these expectations. For studies where the replicates are entirely technical in nature, the standard deviation of the log-cpm decreases steadily as a function of the mean (Figure 1a). For studies where the replicates are genetically identical mice, the standard deviation asymptotes at a moderate level corresponding to a biological CV of about 10% (Figure 1b). Studies where the replicates are unrelated human individuals show greater biological variation. For these studies, the standard deviation asymptotes early and at a relatively high level (Figure 1d).

We conclude that log-cpm values generally show a smoothly decreasing mean-variance trend with count size, and that the log-cpm transformation roughly de-trends the variance of the RNA-seq counts as a function of count size for genes with larger counts.

### Using log-cpm in a limma pipeline

A simple approach to analyzing RNA-seq data would be to input the log-cpm values into a well-established microarray analysis pipeline such as that provided by the limma software package [3,9]. This would be expected to behave well if the counts were all reasonably large, but it ignores the mean-variance trend for lower counts. The microarray pipeline should behave better if modified to include a mean-variance trend as part of the variance modeling. We have therefore modified the empirical Bayes procedure of the limma package so that the gene-wise variances are squeezed towards a global mean-variance trend curve instead of towards a constant pooled variance. This is similar in principle to the procedure proposed by Sartor *et al*. [36] for microarray data, except that we model the trend using a regression spline and our implementation allows for the possibility of missing values or differing residual degrees of freedom between genes. We call this strategy *limma-trend*, whereby the log-cpm values are analyzed as for microarray data but with a trended prior variance. For comparison, the more naive approach without the mean-variance trend will be called *limma-notrend*.

### voom: variance modeling at the observation-level

The limma-trend pipeline models the variance at the gene level. However, in RNA-seq applications, the count sizes may vary considerably from sample to sample for the same gene. Different samples may be sequenced to different depths, so different count sizes may be quite different even if the cpm values are the same. For this reason, we wish to model the mean-variance trend of the log-cpm values at the individual observation level, instead of applying a gene-level variability estimate to all observations from the same gene.

Our strategy is to estimate non-parametrically the mean-variance trend of the logged read counts and to use this mean-variance relationship to predict the variance of each log-cpm value. The predicted variance is then encapsulated as an inverse weight for the log-cpm value. When the weights are incorporated into a linear modeling procedure, the mean-variance relationship in the log-cpm values is effectively eliminated.

**Figure 2:**
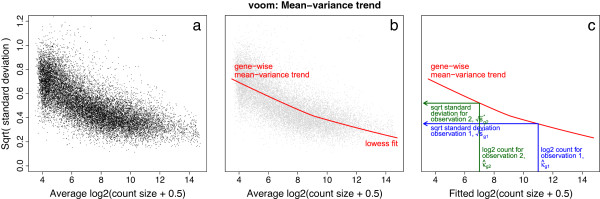
**voom mean-variance modeling.(a)** Gene-wise square-root residual standard deviations are plotted against average log-count. **(b)** A functional relation between gene-wise means and variances is given by a robust LOWESS fit to the points. **(c)** The mean-variance trend enables each observation to map to a square-root standard deviation value using its fitted value for log-count. LOWESS, locally weighted regression.

A technical difficulty is that we want to predict the variances of individual observations although there is, by definition, no replication at the observational level from which variances could be estimated. We work around this inconvenience by estimating the mean-variance trend at the gene level, then interpolating this trend to predict the variances of individual observations (Figure 2).

The algorithm proceeds as follows. First, gene-wise linear models are fitted to the normalized log-cpm values, taking into account the experimental design, treatment conditions, replicates and so on. This generates a residual standard deviation for each gene (Figure 2a). A robust trend is then fitted to the residual standard deviations as a function of the average log-count for each gene (Figure 2b).

Also available from the linear models is a fitted value for each log-cpm observation. Taking the library sizes into account, the fitted log-cpm for each observation is converted into a predicted count. The standard deviation trend is then interpolated to predict the standard deviation of each individual observation based on its predicted count size (Figure 2c). Finally, the inverse squared predicted standard deviation for each observation becomes the weight for that observation.

The log-cpm values and associated weights are then input into limma’s standard differential expression pipeline. Most limma functions are designed to accept quantitative weights, providing the ability to perform microarray-like analyses while taking account of the mean-variance relationship of the log-cpm values at the observation level.

### voom and limma-trend control the type I error rate correctly

We have found that voom and limma-trend, especially voom, perform well and produce *P* values that control error rates correctly over a wide range of simulation scenarios. For illustration, we present results from simulations in which read counts were generated under the same NB model as assumed by a number of existing RNA-seq analysis methods. These simulations should represent the ideal for the NB-based methods. If the normal-based methods can give performance comparable to or better than count-based methods in these simulations, then this is strong evidence that they will be competitive across a wide range of data types.

Six RNA-seq count libraries were simulated with counts for 10,000 genes. The first three libraries were treated as group 1 and the others as group 2. The distribution of cpm values for each library was simulated to match the distribution that we observed for a real RNA-seq dataset from our own practice. The NB dispersion *ϕ* was set to decrease on average with expected count size, asymptoting to 0.2 squared for large counts. This degree of biological variation is representative of what we observe for real RNA-seq data, being larger than we typically observe between genetically identical laboratory mice but less than we typically see between unrelated human subjects (Figure 1). An individual dispersion *ϕ* was generated for each gene around the trend according to an inverse chi-square distribution with 40 degrees of freedom. The voom mean-variance trend for one such simulated dataset is shown in Figure 1c. It can be seen from Figure 1 that the simulated dataset is intermediate between the mouse data (Figure 1b) and the human data (Figure 1d) both in terms of the absolute size of the dispersions and in terms of heterogeneity of the dispersions between genes.

We found that variation in sequencing depth between libraries had a noticeable impact on some RNA-seq analysis methods. Hence all the simulations were repeated under two library size scenarios, one with the same sequencing depth for all six libraries and one with substantial variation in sequencing depth. In the equal size scenario, all libraries were simulated to contain 11 million reads. In the unequal size scenario, the odd-numbered libraries were simulated to have a sequence depth of 20 million reads while the even-numbered libraries had a sequence depth of 2 million reads. Hence the same total number of reads was simulated in this scenario but distributed unevenly between the libraries.

**Figure 3:**
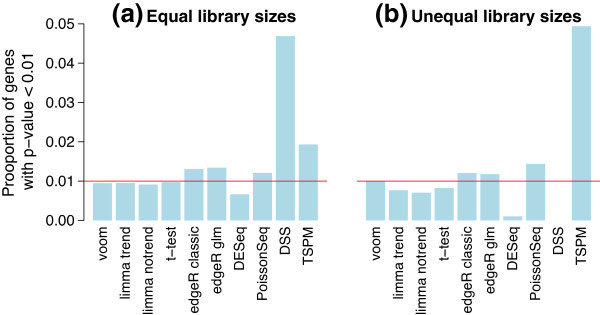
**Type I error rates in the absence of true differential expression.** The bar plots show the proportion of genes with *P*<0.01 for each method **(a)** when the library sizes are equal and **(b)** when the library sizes are unequal. The red line shows the nominal type I error rate of 0.01. Results are averaged over 100 simulations. Methods that control the type I error at or below the nominal level should lie below the red line.

In the first set of simulations, we examined the ability of voom and limma-trend to control the type I error rate correctly in the absence of any genuine differential expression between the groups. When there are no truly differentially expressed genes, the gene-wise *P* values should follow an approximate uniform distribution. If the type I error rate is controlled correctly, then the expected proportion of *P* values below any cutoff should be less than or equal to the cutoff value. A number of popular RNA-seq analysis methods based on the NB or Poisson distributions were included for comparison. Figure 3 shows results for a *P* value cutoff of 0.01. Results for other cutoffs are qualitatively similar. None of the NB- or Poisson-based methods were found to control the type I error rate very accurately. When the library sizes are equal, the NB and Poisson methods were overly liberal, except for DESeq which is very conservative. When the library sizes are unequal, DSS and DESeq became extremely conservative. By contrast, all the normal-based methods were slightly conservative. voom produces results very close to the nominal type I error rate for both library size scenarios. limma-trend is similar to voom when the library sizes are equal but somewhat conservative when the library sizes are unequal.

**Figure 4:**
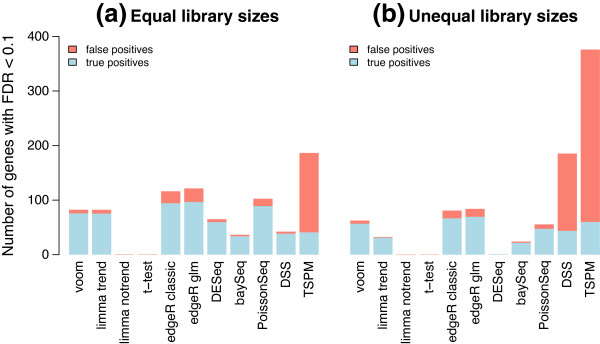
**Power to detect true differential expression.** Bars show the total number of genes that are detected as statistically significant (FDR < 0.1) **(a)** with equal library sizes and **(b)** with unequal library sizes. The blue segments show the number of true positives while the red segments show false positives. 200 genes are genuinely differentially expressed. Results are averaged over 100 simulations. Height of the blue bars shows empirical power. The ratio of the red to blue segments shows empirical FDR. FDR, false discovery rate.

baySeq was not included in the type I error rate comparison because it does not return *P* values. However, the results presented in the next section show that it is relatively conservative in terms of the false discovery rate (FDR) (Figure 4).

To check voom’s conservativeness on real data, we used a set of four replicate libraries from the SEQC Project [37]. All four libraries were Illumina HiSeq 2000 RNA-seq profiles of samples of Ambion’s Human Brain Reference RNA (HBRR) [38]. We split the four libraries into two groups in all possible ways, and tested for differential expression between the two groups for each partition. voom returned no differentially expressed (DE) genes at 5% FDR for six out of the seven possible partitions, indicating good error rate control. The voom mean-variance trend for the SEQC data, using all the libraries rather than the HBRR samples only, is shown in Figure 1a.

### voom has the best power of methods that control the type I error rate

Next we examined the power to detect true differential expression. For the following simulations, 100 randomly selected genes were twofold upregulated in the first group and another 100 were twofold upregulated in the second group. This represents a typical scenario for a functional genomics experiment in which the differential expression effects are large enough to be biologically important but nevertheless sufficiently subtle as to challenge many analysis methods. Figure 4 shows the number of true and false discoveries made by various analysis methods at significance cutoff FDR <0.1. When the library sizes are equal, voom and limma-trend have the next best power after edgeR and PoissonSeq. However, both edgeR and PoissonSeq give empirical FDRs greater than 0.1, confirming the results of the previous section that these methods are somewhat liberal. limma-trend gives an empirical FDR slightly greater than voom but still less than 0.1. With unequal library sizes, voom has the best power except for edgeR while still maintaining a low FDR. TSPM declares by far the most DE genes, but these are mostly false discoveries. DSS also gives a worryingly high rate of false discoveries when the library sizes are unequal. Figures 3 and 4 together show that voom has the best power of those methods that correctly control the type I and FDR error rates.

### voom has the lowest false discovery rate

**Figure 5:**
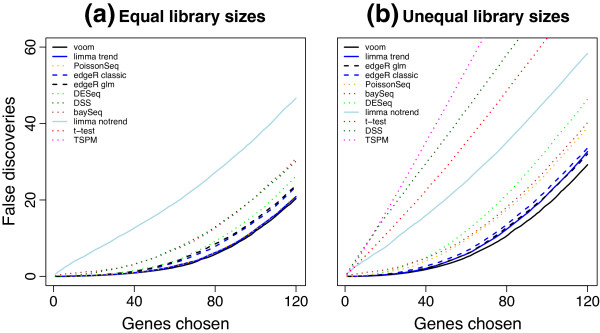
**False discovery rates.** The number of false discoveries is plotted for each method versus the number of genes selected as differentially expressed. Results are averaged over 100 simulations **(a)** with equal library sizes and **(b)** with unequal library sizes. voom has the lowest FDR at any cutoff in either scenario. FDR, false discovery rate.

Next we compared methods from a gene ranking point of view, comparing methods in terms of the number of false discoveries for any given number of genes selected as DE. Methods that perform well will rank the truly DE genes in the simulation ahead of non-DE genes. Genes were ranked by posterior likelihood for baySeq and by *P* value for the other methods. The results show that voom has the lowest FDR at any cutoff (Figure 5). When the library sizes are equal, limma-trend and PoissonSeq are very close competitors (Figure 5a). When the library sizes are unequal, limma-trend and edgeR are the closest competitors (Figure 5b).

**Figure 6:**
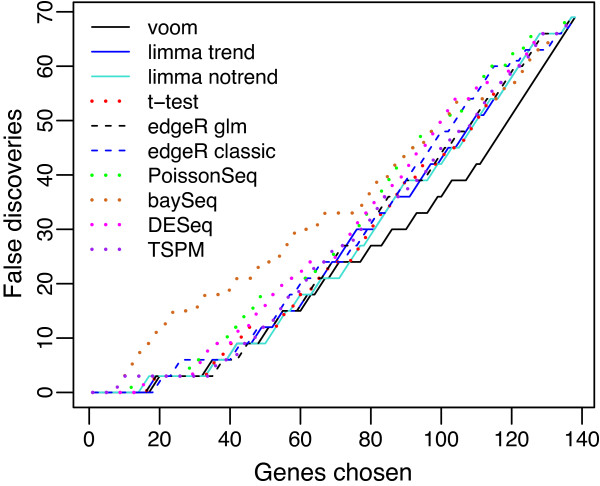
**False discovery rates evaluated from SEQC spike-in data.** The number of false discoveries is plotted for each method versus the number of genes selected as differentially expressed. voom has the lowest false discovery rate overall.

Next we compared FDRs using spike-in control transcripts from the SEQC project [39]. The data consists of eight RNA-seq libraries, in two groups of four. A total of 92 artificial control transcripts were spiked-in at different concentrations in such a way that three quarters of the transcripts were truly DE and the remaining quarter were not. To make the spike-ins more like a realistic dataset, we replicated the counts for each of the 23 non-DE transcripts three times. That is, we treated each non-DE transcript as three different transcripts. This resulted in a dataset of 138 transcripts with half DE and half non-DE. Figure 6 is analogous to Figure 5 but using the spike-in data instead of simulated data. voom again achieved the lowest FDR, with edgeR and the other limma methods again being the closest competitors (Figure 6).

### voom and limma-trend are faster than specialist RNA-seq methods

**Figure 7:**
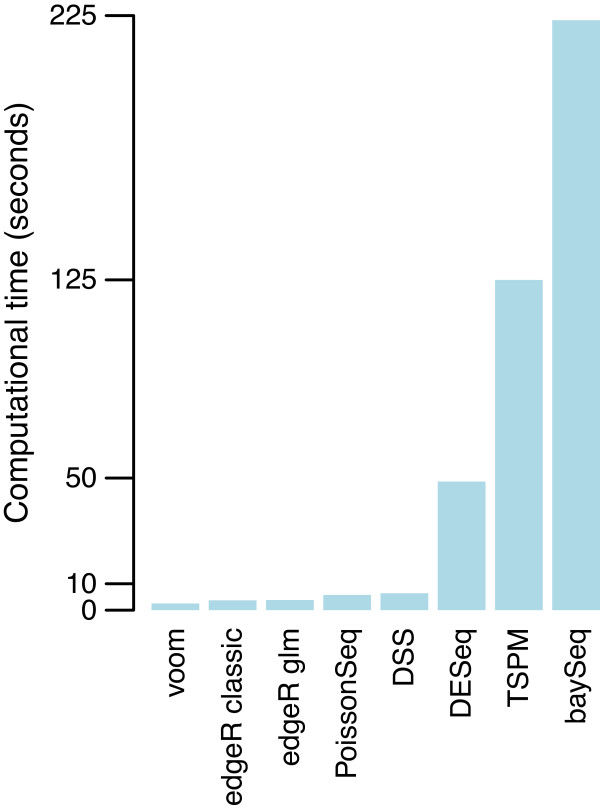
**Computing times of RNA-seq methods.** Bars show time in seconds required for the analysis of one simulated dataset on a MacBook laptop. Methods are ordered from quickest to most expensive.

The different statistical methods compared varied considerably in computational time required, with DESeq, TSPM and baySeq being slow enough to limit the number of simulations that were done. voom is easily the fastest of the methods compared, with edgeR-classic next fastest (Figure 7).

### RNA-seq profiles of male and female Nigerian individuals

So far we have demonstrated the performance of voom on RNA-seq datasets with small numbers of replicate libraries. To demonstrate the performance of voom on a heterogeneous dataset with a relatively large number of replicates and a high level of biological variability, we compared males to females using RNA-seq profiles of lymphoblastoid cell lines from 29 male and 40 female unrelated Nigerian individuals [40]. Summarized read counts and gene annotation are provided by the Bioconductor tweeDEseqCountData package [41]. Figure 1d shows the voom mean-variance trend of this dataset.

voom yielded 16 genes upregulated in males and 43 upregulated in females at 5% FDR. As expected, most of the top differentially expressed genes belonged to the X or Y sex chromosomes (Table 1). The top gene is XIST, which is a key player in X inactivation and is known to be expressed at meaningful levels only in females.

**Table 1:** Top 16 genes differentially expressed between males and females in the Nigerian data

**Ensembl ID**	**Symbol**	**Chr**	**logFC**	**AveExpr**	***t***	***P*** **value**	**FDR**	***B***
ENSG00000229807	XIST	X	-9.815	3.8084	-36.4	7.03e-48	1.19e-43	74.8
ENSG00000099749	CYorf15A	Y	4.251	0.3146	28.3	1.25e-40	1.05e-36	68.2
ENSG00000157828	RPS4Y2	Y	3.281	3.3081	26.5	9.38e-39	5.27e-35	72.6
ENSG00000233864	TTTY15	Y	4.897	-0.5538	25.9	4.31e-38	1.82e-34	64.0
ENSG00000131002	CYorf15B	Y	5.440	-0.1710	23.2	4.81e-35	1.62e-31	60.0
ENSG00000198692	EIF1AY	Y	2.398	2.6806	20.5	1.09e-31	3.07e-28	58.6
ENSG00000165246	NLGN4Y	Y	5.330	-0.4916	19.7	1.26e-30	3.03e-27	52.4
ENSG00000213318	RP11-331F4.1	16	4.293	2.2654	19.3	4.44e-30	9.34e-27	54.1
ENSG00000129824	RPS4Y1	Y	2.781	4.7118	17.6	9.28e-28	1.74e-24	51.5
ENSG00000183878	UTY	Y	1.878	2.7430	16.6	2.88e-26	4.85e-23	47.7
ENSG00000012817	KDM5D	Y	1.470	4.7046	14.9	1.45e-23	2.22e-20	42.6
ENSG00000146938	NLGN4X	X	4.472	-0.7801	14.8	2.09e-23	2.94e-20	38.9
ENSG00000243209	AC010889.1	Y	2.528	-0.0179	14.5	5.48e-23	7.11e-20	37.9
ENSG00000067048	DDX3Y	Y	1.671	5.3077	13.4	3.05e-21	3.67e-18	37.5
ENSG00000006757	PNPLA4	X	-0.988	2.5341	-10.4	4.78e-16	5.38e-13	25.7
ENSG00000232928	RP13-204A15.4	X	1.434	3.2506	10.3	1.02e-15	1.08e-12	25.2

The table shows output from the limma topTable function. As well as gene ID and symbol, columns give chromosome location (Chr), log_2_-fold-change (logFC), average log_2_ expression (AveExpr), moderated *t*, *P* value, false discovery rate and *B*-statistic for each gene. The *B*-statistic is the posterior log-odds of differential expression.

We examined 12 particular genes that are known to belong to the male-specific region of chromosome Y [42,43]. A ROAST gene set test confirmed that these genes collectively are significantly upregulated in males (*P*=0.0001). A CAMERA gene set test was even more convincing, confirming that these genes are significantly more upregulated in males than are other genes in the genome (*P*=2×10^-28^).

**Figure 8:**
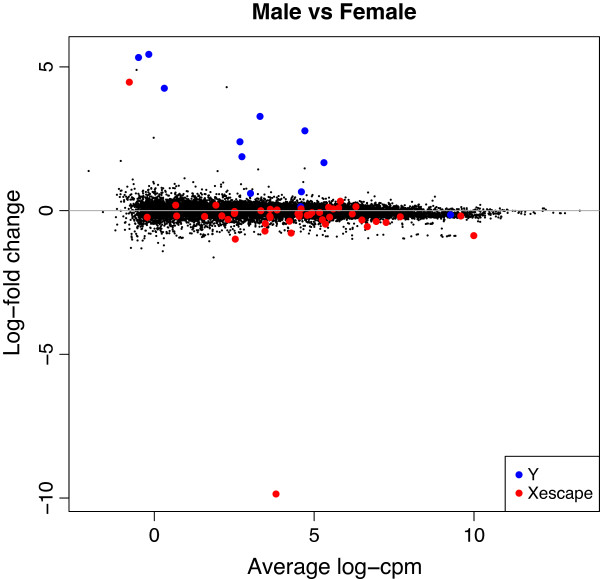
**MA plot of male vs female comparison with male- and female-specific genes highlighted.** The MA plot was produced by the limma plotMA function, and is a scatterplot of log-fold-change versus average log-cpm for each gene. Genes on the male-specific region of the Y chromosome genes are highlighted blue and are consistently upregulated in males, while genes on the X chromosome reported to escape X inactivation are highlighted red and are generally down in males. log-cpm, log-counts per million.

We also examined 46 X chromosome genes that have been reported to escape X inactivation [43,44]. These genes were significantly upregulated in females (ROAST *P*=0.0001, CAMERA *P*=10^-10^). The log-fold-changes for the X and Y chromosome genes involved in the gene set tests are highlighted on an MA plot (Figure 8).

Note that these gene set testing approaches are not available for any of the count-based approaches to differential expression. If a count-based method had been used to assess differential expression, we could still have examined whether sex-linked genes were highly ranked among the differentially expressed genes, but we could not have undertaken any formal statistical test for enrichment of this signature while accounting for inter-gene correlation. On the other hand, the voom expression values and weights are suitable for input into the ROAST and CAMERA procedures without any further processing.

### Development stages of *Drosophila melanogaster*

Like edgeR-glm, but unlike most other analysis tools, voom and limma-trend offer full-featured linear modeling for RNA-seq data, meaning that they can analyze arbitrary complex experiments. The possibilities of linear modeling are so rich that it is impossible to select a representative example. voom and limma could be used to analyze any gene-level RNA-seq differential expression experiment, including those with multiple experimental factors [34]. Here we give a novel analysis illustrating the use of quadratic regression to analyze a time-course study.

RNA-seq was used to explore the developmental transcriptome of *Drosophila melanogaster*[45]. RNA-seq libraries were formed from whole-animal samples to represent a large number of distinct developmental stages. In particular, samples were collected from embryonic animals at equi-spaced development stages from 2 hours to 24 hours in 2-hour intervals. Here we analyze the 12 RNA-seq libraries from these embryonic stages. We sought to identify those genes that are characteristic of each embryonic stage. In particular we wished to identify, for each embryonic stage, those genes that achieve their peak expression level during that stage.

**Figure 9:**
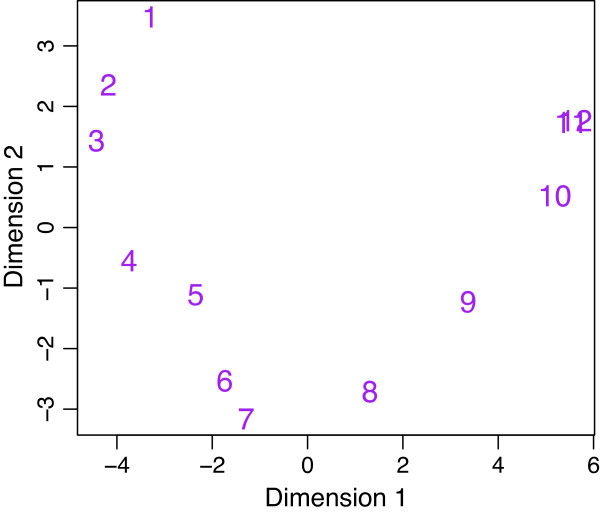
**Multidimensional scaling plot of*Drosophila melanogaster* embryonic stages.** Distances are computed from the log-cpm values. The 12 successive embryonic developmental stages are labeled 1 to 12, from earliest to latest.

As all the samples are from distinct stages, there are no replicate libraries in this study. To estimate variances we utilized the fact that gene expression should for most genes vary smoothly over time. A multidimensional scaling plot of log-cpm values shows the gradual change in gene expression during embryonic development, with each stage intermediate in expression profile between the stages before and after (Figure 9). We used gene-wise linear models to fit a quadratic trend with time to the log-cpm values for each gene. These quadratic trends will not match all the intricacies of gene expression changes over time but are sufficient to model the major trends. The voom mean-variance trend for this data is shown in Figure 1e.

**Figure 10:**
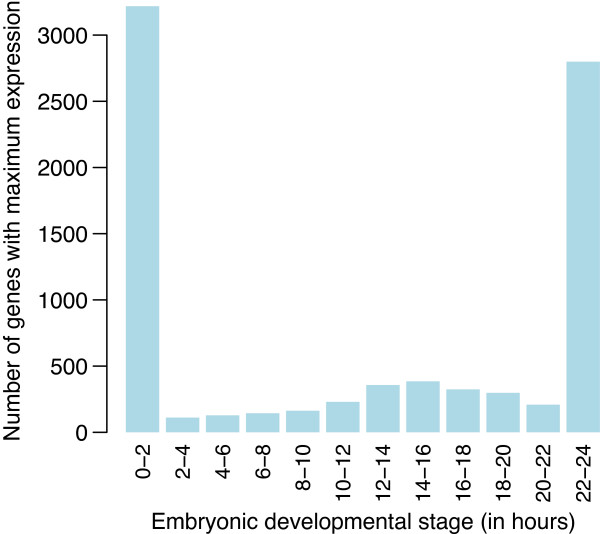
**Number of genes associated with each*Drosophila melanogaster* embryonic stage.** The number of genes whose peak estimated expression occurs at each of the stages is recorded.

**Figure 11:**
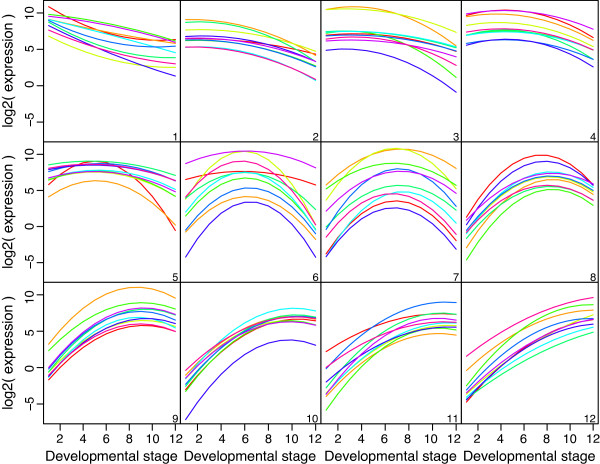
**Expression trends for genes that peak at each*Drosophila melanogaster* embryonic stage.** Panels **(1)** to **(12)** correspond to the 12 successive developmental stages. Each panel displays the fitted expression trends for the top ten genes that achieve their peak expression during that stage. In particular, panel **(1)** shows genes that are most highly expressed at the first stage and panel **(12)** shows genes most highly expressed at the last stage. Panels **(7)** and **(8)** are notable because they show genes with marked peaks at 12–14 hours and 14–16 hours respectively.

Out of 14,869 genes that were expressed during embryonic development, 8,366 showed a statistically significant trend at 5% FDR using empirical Bayes *F*-tests. For each differentially expressed gene, we identified the embryonic stage at which the fitted quadratic trend achieved its maximum value. This allowed us to associate each significant gene with a particular development stage (Figure 10). Most genes peaked at the first or last stage (Figure 10), indicating smoothly decreasing or increasing trends over time (Figure 11, panels 1 and 12). Genes peaking at the first embryonic stage tended to be associated with the cell cycle. Genes peaking at the last stage tended to be associated with precursor metabolites and energy, the oxidation-reduction process and with metabolic pathways.

Genes peaking at intermediate stages have expression trends with an inverse-U shape (Figure 11, panels 2–11). There was a substantial set of genes with peak activity between 12–16 hours of embryonic development (Figure 10), suggesting some important developmental change occurring during this period requiring the action of special-purpose genes. Indeed, gene ontology analysis of the genes associated with this period showed that anatomical structure morphogenesis was the most significantly enriched biological process. Other leading terms were organ morphogenesis and neuron differentiation.

This analysis demonstrates a simple but effective means of identifying genes that have a particular role at each developmental stage.

## Discussion

This article follows the common practice of examining differential expression on a gene-wise basis. Our preferred practice is to count the total number of reads overlapping annotated exons for each genes. While this approach does not allow for the possibility that different isoforms of the same gene may be differentially expressed in different directions, it does provide a statistically robust gene-level analysis even when the sequencing depths are quite modest. The relevance of gene-level analyses is also supported by recent surveys of transcription, which have shown that each gene tends to have a dominant isoform that accounts for far more of the total expression for that gene than any of the remaining isoforms [46,47]. The voom analysis can also be conducted at the exon level instead of at the gene level as an aid to detecting alternative splicing between the treatment groups.

In this article, voom has been applied to log-cpm values. voom can work, however, just as easily with logged rpkm values in place of log-cpm, because the precision weights are the same for both measures. If the genomic length of each gene is known, then the log-cpm values output by voom can be converted to log-rpkm by subtracting the log2 gene length in kilobases. The downstream analysis is unchanged and will yield identical results in terms of differentially expressed genes and estimated fold-changes.

This article has shown that a normal-based analysis of RNA-seq read count data performs surprisingly well relative to methods that use special-purpose count distributions. The motivation for examining normal-based methods was to open up access to a range of microarray-like analysis tools based on the normal distribution. From this point of view, the normal-based methods only need to perform comparably to the count-based methods in terms of power and FDR control in order to be a success. Our comparisons suggest not only that this is so, but that the normal-based methods actually have a performance advantage. We found voom to be the best performer across our simulations and comparisons, and even the simpler limma-trend method performed equal or better than the count-based methods. voom and limma-trend perform almost equally when the library sizes are equal, but voom has the advantage when the library sizes are unequal. The best performing count-based methods were edgeR and PoissonSeq, although neither of those methods controlled the type I error rate at the nominal level, both being somewhat liberal. The performance advantage of voom over many of the count-based methods was quite substantial in our simulations, despite the simulations being conducted under the same NB distributional assumptions as made by a number of existing methods. Other simulation scenarios would tend to increase voom’s advantage. For example, it would be at least as scientifically reasonable to assume that the true expression levels for each gene follow a log-normal distribution between replicates instead of a gamma distribution, and such an assumption would tend to improve the performance of voom relative to edgeR, DESeq, baySeq and DSS. In general, voom makes fewer distributional assumptions than do competing methods and can therefore be expected to perform robustly across a range of scenarios. This study presented simulations with equal library sizes between replicates, and also explored the sensitivity of the methods to unequal library sizes. In our experience markedly unequal library sizes can arise in real RNA-seq experiments for a variety of reasons. One scenario is when an experiment is conducted in stages and samples sequenced at a later time have a much higher sequencing depth. Other possible scenarios occur when technical replicates are combined for a subset of samples or when DNA samples are multiplexed onto a sequencing lane in unequal quantities. Some of the NB-based analysis methods become very conservative or showed very poor FDR control when the library sizes were unequal. In contrast, voom shows consistent performance in all scenarios.

The worst performer in our simulation was TSPM, presumably because we have simulated from NB distributions, which have quadratic mean-variance relationships, whereas TSPM assumes a linear mean-variance relationship [25]. The second worst performer was the ordinary *t*-test. This shows that traditional statistical methods cannot be reliably applied to genomic data without borrowing strength between genes. The third worst performer was limma-notrend, showing that the mean-variance trend in the log-cpm values cannot be ignored.

To examine sensitivity of the results to the shape of the dispersion distribution, we repeated all the simulations using a log-normal distribution for the gene-wise dispersions instead of an inverse chi-square distribution. The two distributions were chosen to have the same mean and variance on the log-scale. The results were virtually unchanged from those shown in Figures 3, 4 and 5, showing that the shape of the dispersion distribution is not a major determination of performance. This agrees with a similar conclusion in Wu *et al*. [22].

It may seem surprising at first that voom should perform so well even though it ignores the discrete integer nature of the counts. We think there are several possible reasons for this. First, the parametric advantages of the Poisson or NB distributions are mitigated by the fact that the observed mean-variance relationship of RNA-seq data does not perfectly match the theoretical mean-variance relationships inherent in these distributions. While the quadratic mean-variance relationship of the NB distribution captures most of the mean-variance trend, the NB dispersion still shows a non-ignorable trend with gene abundance [13,19,34]. This means that the mean-variance relationship still has to be estimated non-parametrically, at least in part.

Second, voom is more precise than previous methods in terms of its treatment of the mean-variance trend. While several previous methods fit a semi-parametric trend to the variances or to the NB dispersions [13,19,23,34], the trend has always been used to estimate gene-level model parameters. This ignores the fact that different counts for the same gene may vary substantially in size, meaning that the trend should be applied differently to different observations. This consideration becomes more critical when different RNA samples are sequenced to different depths.

Third, the use of normal models gives voom access to tractable empirical Bayes distribution theory [3], facilitating reliable estimation of the Bayesian hyperparameters and exact small sample distributions for the test statistics. Amongst other things this facilitates accurate estimate of the prior degrees of freedom determining the optimal amount of squeezing to be applied to the variances.

Fourth, the use of normal distribution approximations in conjunction with variance modeling is partly supported by generalized linear model theory. Rao’s score test [48] for a covariate in a generalized linear model is essentially equivalent to the normal theory test statistic, provided that the mean-variance function is correctly estimated and incorporated into appropriate precision weights [49]. Score tests have similar performance to likelihood ratio tests when the null hypothesis is true or when the changes being detected are relatively small.

Some of the count-based methods have been criticized as being sensitive to outlier counts [28]. The voom and limma-trend methods inherit good robustness properties from the normal-based procedures in limma [28]. If necessary, they can be made extremely robust to outliers and hypervariable genes using the robust empirical Bayes options of the limma package [50].

In addition to performance results, voom has a number of qualitative advantages over the count-based methods. It is fast and convenient. It allows RNA-seq and microarray data to be analyzed in closely comparable ways, which may be an attraction for analysts comparing results from the two technologies. It gives access to a wealth of statistical methods developed for microarrays, including for example the gene set testing methods demonstrated on the Nigerian dataset.

## Conclusions

voom performs as well or better than existing RNA-seq methods, especially when the library sizes are unequal. It is moreover faster and more convenient, and converts RNA-seq data into a form that can be analyzed using similar tools as for microarrays.

## Materials and methods

### Log-counts per million

We assume that an experiment has been conducted to generate a set of *n* RNA samples. Each RNA sample has been sequenced, and the sequence reads have been summarized by recording the number mapping to each gene. The RNA-seq data consist therefore of a matrix of read counts *r*_*gi*_, for RNA samples *i*=1 to *n*, and genes *g*=1 to *G*. Write *R*_*i*_ for the total number of mapped reads for sample *i*:

$$R_{i}=\overset{G}{\underset{g=1}{\sum }}r_{\text{gi}}$$

We define the log-counts per million (log-cpm) value for each count as:

$$y_{\text{gi}}=\underset{2}{\log}\left(\frac{r_{\text{gi}}+0.5}{R_{i}+1.0}\times 10^{6}\right)$$

The counts are offset away from zero by 0.5 to avoid taking the log of zero, and to reduce the variability of log-cpm for low expression genes. The library size is offset by 1 to ensure that (*r*_*gi*_+0.5)/(*R*_*i*_+1) is strictly less than 1 as well as strictly greater than zero.

### Delta rule for log-cpm

Write *λ*=*E*(*r*) for the expected value of a read count given the experimental conditions, and suppose that:

$$\text{var}(r)=\lambda +\phi \lambda^{2}$$

where *ϕ* is a dispersion parameter. If *r* is large, then the log-cpm value of the observation is:

$$y\approx \underset{2}{\log}(r)-\underset{2}{\log}(R)+6\underset{2}{\log}(10)$$

where *R* is the library size. Note that the analysis is conditional on *R*, so *R* is treated as a constant. It follows that var(*y*)≈var(log2(*r*). If *λ* also is large, then:

$$\underset{2}{\log}(r)\approx \lambda +\frac{r-\lambda }{\lambda }$$

by Taylor’s theorem [51], so:

$$\text{var}(\;y)\approx \frac{\text{var}(r)}{\lambda^{2}}=\frac{1}{\lambda }+\mathrm{\phi .}$$

### Linear models

This article develops differential expression methods for RNA-seq experiments of arbitrary complexity, for example experiments with multiple treatment factors, batch effects or numerical covariates. As has been done previously [3,7,8,34], we use linear models to describe how the treatment factors are assigned to the different RNA samples. We assume that:

$$E(\;y_{\text{gi}})=\mu_{\text{gi}}=x_{i}^{T}\beta_{g}$$

where *x*_*i*_ is a vector of covariates and *β*_*g*_ is a vector of unknown coefficients representing log2-fold-changes between experimental conditions. In matrix terms:

$$E(\;y_{g})=X\beta_{g}$$

where *y*_*g*_ is the vector of log-cpm values for gene *g* and *X* is the design matrix with the *x*_*i*_ as rows. Interest centers on testing whether one or more of the *β*_*gj*_ are equal to zero,

### voom variance modeling

The above linear model is fitted, by ordinary least squares, to the log-cpm values *y*_*gi*_ for each gene. This yields regression coefficient estimates ${\hat{\beta }}_{g}$, fitted values ${\hat{\mu }}_{\text{gi}}=x_{i}^{T}{\hat{\beta }}_{g}$ and residual standard deviations *s*_*g*_.

Also computed is the average log-cpm ${\bar{y}}_{g}$ for each gene. The average log-cpm is converted to an average log-count value by:

$$\tilde{r}={\bar{y}}_{g}+\underset{2}{\log}(\tilde{R})-\underset{2}{\log}(10^{6})$$

where $\tilde{R}$ is the geometric mean of the library sizes plus one.

To obtain a smooth mean-variance trend, a LOWESS curve is fitted to square-root standard deviations $s_{g}^{1/2}$ as a function of mean log-counts $\tilde{R}$ (Figure 2a,b). Square-root standard deviations are used because they are roughly symmetrically distributed. The LOWESS curve [52] is statistically robust [53] and provides a trend line through the majority of the standard deviations. The LOWESS curve is used to define a piecewise linear function lo() by interpolating the curve between ordered values of $\tilde{R}$.

Next the fitted log-cpm values ${\hat{\mu }}_{\text{gi}}$ are converted to fitted counts by:

$${\hat{\lambda }}_{\text{gi}}={\hat{\mu }}_{\text{gi}}+\underset{2}{\log}(R_{i}+1)-\underset{2}{\log}(10^{6}).$$

The function value $\text{lo}({\hat{\lambda }}_{\text{gi}})$ is then the predicted square-root standard deviation of *y*_*gi*_.

Finally, the voom precision weights are the inverse variances $w_{\text{gi}}=\text{lo}{\left({\hat{\lambda }}_{\text{gi}}\right)}^{-4}$ (Figure 2c). The log-cpm values *y*_*gi*_ and associated weights *w*_*gj*_ are then input into limma’s standard linear modeling and empirical Bayes differential expression analysis pipeline.

### Gene set testing methods

ROAST [7] and CAMERA [8] are gene set testing procedures that assess changes in the overall expression signature defined by a set of genes. ROAST [7] is a self-contained test that assesses differential expression of the gene set without regard to genes not in the set. CAMERA [8] is a competitive test that assesses differential expression of the gene set relative to all other genes on the array. Both procedures offer considerable flexibility as they have the ability to test the association of a genomic pathway or gene set signature with quite general treatment comparisons or contrasts defined in the context of a microarray linear model. We have adapted both methods to make use of quantitative weights as output by voom. The revised methods are implemented in the functions roast() and camera() of the limma software package.

### Normalization

The log-cpm values are by definition normalized for sequencing depth. Other normalization steps can optionally be done. The library sizes *R*_*i*_ can be scale normalized to adjust for compositional differences between the RNA-seq libraries [54]. This produces normalized library sizes $R_{i}^{*}$ that can be used in place of *R*_*i*_ in the voom pipeline. Alternatively, between-array normalization methods developed for single channel microarray data, such as quantile or cyclic LOESS, can be are applied to the log-cpm values.

### Simulations

The simulations were designed to generate data with characteristics similar to real data that we analyze in our own practice. First a set of baseline expression values was generated representing the relative proportion of counts expected to arise from each gene. These proportions were translated into expected count sizes by multiplying by library size, and then multiplied by true fold-changes as appropriate. Counts were then generated following a NB distribution with the specified mean and dispersion for each observation.

The distribution of baseline values was chosen to match that from RNA-seq experiments conducted at our institution. Specifically we used the goodTuringProportions function of the edgeR package [12], which implements the Good-Turing algorithm [55], to predict the true proportion of total RNA attributable to each gene. We ran this function on a number of different libraries, pooled the predicted proportions and formed a smoothed distribution function. The baseline proportions for the simulations were then generated to follow this distribution.

The NB dispersions were generated as follows. The trend in the dispersions was set to be *ψ*_*gi*_ with:

$$\psi_{\text{gi}}^{1/2}=0.2+\lambda_{\text{gi}}^{-1/2}$$

where *λ*_*gi*_ is the expected count size. A modest amount of gene-wise biological variation was generated from an inverse chi-square distribution with 40 degrees of freedom. The individual dispersions were set to be *ϕ*_*gi*_=*ψ*_*gi*_*δ*_*g*_ where $40/\delta_{g}∼\chi_{40}^{2}$.

In an alternative simulation, to investigate sensitivity to the distribution of gene-wise dispersions, the *δ*_*g*_ were simulated as log-normal with mean 0 and standard deviation 0.25 on the log-scale. This produces a distribution with a similar CV as for the inverse chi-square simulation.

For each simulated dataset, genes with less than ten reads across all samples were filtered from the analysis. PoissonSeq resets the seed of the random number generator in R, so it was necessary to save and restore the state of the random number generator before and after each call of the main PoissonSeq function.

Complete runnable code that reproduces all the simulations is provided as Additional file 1. See also the voom website [56].

### SEQC data

The SEQC project, also known as MAQC-III, aims to provide a comprehensive study of next-generation sequencing technologies [37]. We analyze here a pilot SEQC dataset consisting of 16 RNA-seq libraries in four groups. The full SEQC data including the 16 libraries analyzed here will become available as GEO series [GEO:GSE47792] when the main SEQC article is published in 2014. In the meantime, the aligned and summarized read counts for the pilot libraries needed to repeat the analyses in this article are available from the voom webpage [56].

The groups are labeled A–D and are closely analogous to the similarly labeled RNA samples used in the earlier microarray quality control study [57]. Libraries in group A are profiles of Stratagene’s Universal Human Reference RNA (UHRR) with the addition of RNA from Ambion’s ERCC ExFold RNA spike-in mix 1 (Mix 1). Libraries in group B are profiles of Ambion’s Human Brain Reference RNA (HBRR) with added RNA from Ambion’s ERCC ExFold RNA spike-in mix 2 (Mix 2). RNA samples in groups C and D are mixtures of A and B in the proportions 75:25 and 25:75, respectively. An Illumina HiSeq 2000 was used to create a FastQ file of paired-end sequence reads for each sample. The library size for each sample varied from 5.4 to 8.0 million read pairs. Fragments were mapped to the National Center for Biotechnology Information’s Build 37.2 of the human genome using the Subread aligner [58]. Fragment counts were summarized by Entrez Gene ID using the featureCounts function [59] of version 1.8.2 of the Bioconductor package Rsubread [60]. Fragments with both end reads mapped successfully contributed one count if the fragment overlapped any annotated exon for that gene. Fragments for which only one read mapped successfully contributed half a count if that read overlapped an exon. The summarized read count data is available from the voom webpage [56].

The voom mean-variance trend shown in Figure 1a was obtained from all 16 libraries, treated as four groups. Genes were filtered out if they failed to achieve cpm >1 in at least four libraries, and the remaining log-cpm values were quantile normalized between libraries [61].

The comparison between technical replicates to check the type I error rate control used only the four group B libraries. Genes were filtered out if they failed to achieve a cpm >1 in at least two libraries and the log-cpm values for the 16,745 remaining genes were quantile normalized. Samples were separated into all possible two-versus-two and three-versus-one combinations and a limma analysis using voom weights was carried out for each partition.

The false discovery rate analysis was conducted on the spike-in transcripts only. ERCC Mixes 1 and 2 contain 92 transcripts spiked in at different concentrations. For this analysis, fragments were mapped to the known sequences of the spiked-in transcripts using Subread. The experiment is designed so that 23 transcripts have the same concentration in Mix 1 and Mix 2. The remaining transcripts were spiked-in in such a way that 23 transcripts are fourfold more abundant in Mix 1, 23 are 1.5 higher in Mix 2 and 23 are twofold higher in Mix 2. A majority of the spike-in transcripts data are DE. We replicated the counts for each of the 23 non-DE transcripts three times, so that each non-DE transcript was treated as three different transcripts. This resulted in a dataset of 138 transcripts, half DE and half non-DE. Our analysis used read counts for the spike-in transcripts only. TMM-scale normalization [54] was used for all the analysis methods, except for DESeq and PoissonSeq, which have their own built-in normalization methods. No transcripts were filtered, except by PoissonSeq as its standard analysis includes the removal of probes with low counts. The genes that were filtered out by PoissonSeq were re-introduced to the end of the gene ranking, ordered from largest mean count to lowest mean count.

### Lymphoblastoid cell lines from Nigerian individuals

As part of the International HapMap Project, RNA samples were obtained from lymphoblastoid cell lines derived from 69 unrelated Nigerian individuals including 29 males and 40 females [40]. Sequencing was performed using an Illumina Genome Analyzer II. Read counts, summarized by Ensembl gene, and transcript annotations were obtained from version 1.0.9 of the tweeDEseqCountData Bioconductor package [43], specifically from the data objects pickrell1, annotEnsembl63 and genderGenes. Genes were filtered if they failed to achieve a cpm value of 1 in at least 20 libraries. Library sizes were scale-normalized by the TMM method [54] using edgeR software [12] prior to the voom analysis.

### Development stages of *Drosophila melanogaster*

RNA-seq was used to explore the developmental transcriptome of *D. melanogaster*[45]. Mapped read counts are available from the ReCount project [62]. Specifically the pooled version of the modencodefly dataset from the ReCount website [63] provides read counts summarized by Ensembl 61 gene IDs for 30 whole-animal biological samples. We discarded the larval, pupal and adult stages and kept only the 12 embryonic samples. Genes were retained in the analysis if they achieved cpm >1 for any embryonic stage. Effective library sizes were estimated by TMM scale-normalization [54] using edgeR software [12] prior to the voom analysis.

Gene ontology analysis used the GOstats software package [64] and version 2.9.0 of the org.Dm.eg.db annotation package [65]. All GO terms mentioned in the Results section had Fisher’s exact test *P* values less than 10^-10^.

### C57BL/6J and DBA/2J inbred mouse strains

An RNA-seq experiment was carried out to detect differential striatal gene expression between the C57BL/6J (B6) and DBA/2J (D2) inbred mouse strains [66]. Profiles were made for 10 B6 and 11 D2 mice. Mapped read counts summarized by Ensembl 61 gene IDs were downloaded as the bottomly dataset from the ReCount website [63]. Genes were filtered out if they failed to achieve cpm >1 in at least four libraries and the remaining log-cpm values were quantile normalized. The limma-voom analysis compared the two strains and included a batch effect correction for the Illumina flow cell in which each sample was sequenced. The voom mean-variance trend is shown in Figure 1b.

### Software

The results presented in this article were obtained using R version 3.0.0 and the software packages limma 3.16.2, edgeR 3.2.3, baySeq 1.14.1, DESeq 1.12.0, DSS 1.4.0, PoissonSeq 1.1.2 and tweeDEseqCountData 1.0.8. All of these packages are part of the Bioconductor project [67,68], except for PoissonSeq, which is part of the Comprehensive R Archive Network [69]. The TSPM function, dated February 2011, was downloaded in March 2013 from the author’s webpage [70].

The voom methodology proposed in the article is implemented in the voom function of the limma package. The limma-trend method was implemented by inputting the log-cpm values from voom into limma’s standard pipeline, with trend=TRUE for the eBayes function. Hence the limma-trend pipeline was the same as that for voom except that weights were not used in the linear modeling step and the trend option was turned on for the empirical Bayes step. The limma package can be installed from the Bioconductor project repository [71].

All the count-based packages were used with the default differential expression pipelines as recommended in the software for each package. For edgeR 3.2.3 the default prior degrees of freedom for squeezing the gene-wise dispersions is 10. Note that this is a change from versions 3.0.X and earlier for which the default had been 20. For DSS the Wald test was used as recommended in the documentation. The DESeq defaults have changed considerably since the original publication. We used the DESeq function estimateDispersions with sharingMode=~maximum~ and fitType=~local~ and conducted tests using nbinomTest.

The different count-based packages implement different methods of compositional normalization [54]. For our simulations, there are no compositional differences between the libraries so there should be no need to estimate compositional normalization factors. For this reason we did not use the calcNormFactors function with edgeR or estimateSizeFactors with DESeq or estNormFactors with DSS. This should tend to improve the performance of the packages and to make them more comparable, as any differences between the packages can be attributed to the statistical procedures rather than to differences between the normalization strategies.

## Abbreviations

CV: coefficient of variation; DE: differentially expressed; FDR: false discovery rate; HBRR: Ambion’s Human Brain Reference RNA; log-cpm: log-counts per million; LOWESS: locally weighted regression; NB: negative binomial; rpkm: reads per kilobase per million; UHRR: Stratagene’s Universal Human Reference RNA.

## Competing interests

The authors declare that they do not have any competing interests.

## Authors’ contributions

CL and GS developed the method and wrote the manuscript. GS implemented the method and CL performed the analyses. YC contributed to the comparative simulation studies and carried out the gene ontology analysis. WS performed read alignment and summarized the SEQC and mouse RNA-seq datasets. All authors read and approved the final manuscript.

## Supplementary Material

Additional file 1 **Simulation code.** R code to reproduce the simulations presented in Figures 3, 4 and 5. Click here for file
